# Evaluation of Clinical Outcomes after Poor-Quality Embryo Transfer and Prognostic Parameters

**DOI:** 10.3390/jcm12196236

**Published:** 2023-09-27

**Authors:** Nezaket Kadioglu, İnci Kahyaoğlu, İskender Kaplanoğlu, Serdar Dilbaz, Yaprak Engin Üstün

**Affiliations:** 1 Department of Obstetrics and Gynecology, University of Yuksek Ihtisas, Ankara 06530, Turkey; 2 Department of Assisted Reproductive Technology, Etlik City Hospital, University of Health Sciences, Ankara 06620, Turkey; mdincikahyaoglu@gmail.com (İ.K.); driskaplan@gmail.com (İ.K.); 3 Department of Assisted Reproductive Technology, Etlik Zübeyde Hanım Women’s Health Training and Research Hospital, University of Health Sciences, Ankara 06620, Turkey; sdilbaz@gmail.com (S.D.); ustunyaprak@gmail.com (Y.E.Ü.)

**Keywords:** in vitro fertilization, gonadotropin dose, live birth rate, poor embryo quality

## Abstract

We aimed to investigate the clinical results following poor-quality embryo transfer and the parameters to foresee the prognosis. In this study, 2123 cycles that had day 3 and day 5 single-fresh embryo with poor-quality embryo transfers and good-quality embryo transfers were compared. The cycles according to transfer day were evaluated by conducting a subgroup analysis. The correlation between all the obtained demographic characteristics, controlled ovarian stimulation parameters, and cycle results were analysed. Clinical pregnancy was established in 53 patients that underwent transfer in the poor-quality embryo group (14.9%). Of these patients, 36 had live birth (live birth rate per clinical pregnancy 67.9%). In cleavage-stage embryos, live birth rates per clinical pregnancy were higher in poor-quality blastocyst transfer. When analysing the factors affecting live births in the poor-quality embryo group, as the total gonadotropin dose increases, the probability of live birth decreases, as in the probability of hCG positivity. In conclusion, although the probability of pregnancy is low, when clinical pregnancy is established, there is a high chance of having a live birth after poor-quality embryo transfers. This could be regarded as an acceptable option in cycles when only poor-quality embryos are available.

## 1. Introduction

Embryo quality is a significant determinant of treatment success via assisted reproductive techniques (ART). The majority of the ART treatment protocols have shifted towards single embryo transfer (SET) and focus on the specification and transfer of good-quality embryos oriented towards the achievement of live birth. Poor-quality embryos are generally not preferred for transfer or cryopreservation because their implantation potential is lower than good-quality embryos. Despite insufficient evidence, it is believed that transfer of poor-quality embryos results in spontaneous abortion or pregnancy loss. On the other hand, a correlation was demonstrated between cleavage-stage embryo morphological score and chromosomal anomalies. Although the possibility of euploidy is higher in embryos with good morphology during the blastocyst stage, the effect of aneuploidy on embryo quality is unclear [1]. Therefore, an embryo with excellent morphology may carry a genetic anomaly; contrarily, an embryo with poor morphology posing the potential to be euploid is not ruled out [2,3]. Although each of the studies carried out in recent years evaluated this differently, it was observed that pregnancies following poor-quality and good-quality embryo transfers produced similar obstetric and neonatal results and embryo quality was not correlated with increased adverse obstetric and neonatal risks [2,3,4,5,6,7,8].

When considering that poor-quality embryo transfer does not affect perinatal results, this may provide a valuable option to couples in the decision-making process. The aim of this study is to investigate the factors affecting clinical results following poor-quality embryo transfer and the presence of parameters to foresee the prognosis.

## 2. Materials and Methods

### 2.1. Study Design

Medical records of all patients undergoing treatment at the Etlik Zübeyde Hanım Training and Research Hospital between September 2007 and September 2021 were examined retrospectively, and fresh single embryo transfer cycles were included in the study. Approval was obtained from Etlik Zübeyde Hanım Training and Research Hospital Ethics Committee (21 April 2022/05).

Included in the study were only the cycles that had day 3 and day 5 single fresh embryo transfers; and patients that underwent two or more embryo transfers, frozen/thawed cycles, patients with cancelled cycles (insufficient response, no oocytes retrieved, absence of sperm, fertilization failure, poor embryo development) as well as day 2, day 4, and day 6 embryo transfers were excluded from the study. Demographic characteristics (age, body mass index (BMI), number of cycle, duration of infertility, anti-mullerian hormone (AMH) level, basal follicle stimulating hormone (FSH), estradiol (E2) level, antral follicle count (AFC), indication of treatment, male age, total progressive motile sperm count (TPMSC) and morphology) and controlled ovarian stimulation parameters (stimulation protocol, duration of stimulation, total gonadotropin dose, trigger protocol, E2 level—endometrial thickness—number of developed follicles on the trigger day, number of retrieved total and mature oocytes, and fertilization rates) of patients carrying inclusion criteria were evaluated.

### 2.2. Ovarian Stimulation, Intracytoplasmic Sperm Injection (ICSI), and Embryo Transfer Procedures

After the evaluation of ovarian reserve markers for each patient, GnRH antagonist, long agonist, or microdose flare-up protocols were analysed. Gonadotropin dosages were personalised according to the age of the patient, basal serum FSH level, AFC, BMI, and adjusted based on ovarian response. Cycles were followed with serial transvaginal ultrasonographic evaluations and measurements of serum E2 levels. When a minimum of three follicles reached an average of 18 mm diameter, human chorionic gonadotropin (hCG) was administered to induce final oocyte maturation. Oocyte pick-up (OPU) procedure was carried out by transvaginal ultrasound about 35.5–36 h after hCG administration. Mature oocytes were inseminated by ICSI method. Embryo transfer was conducted with transabdominal ultrasonography. All the patients were provided with similar luteal phase support starting from the date of oocyte pick-up.

### 2.3. Assessment of Embryo Development

Fertilization of oocytes was evaluated 18–20 h after ICSI upon observing the presence of pronuclei. Day 3 embryos (61–65 h after ICSI) were rated using an embryo scoring system according to the cell count, size, symmetry, and fragmentation rate. A scoring system was formed for day 5 embryos at the blastocyst stage by considering the blastocyst expansion, inner cell mass, and trophectoderm structure. Scoring was carried out from the best (Grade 1) to the worst (Grade 5) according to this criteria [9]. Grade 1 and Grade 2 embryos were classified as good-quality embryos, and Grade 3 and Grade 4 embryos were deemed poor-quality embryos. Grade 5 embryos were not transferred.

The results obtained from patients designated as the study group that underwent poor-quality embryo transfer were compared with the results of patients that had a good-quality embryo transfer. Additionally, whether a difference was present between cycles that underwent day 3 and day 5 embryo transfer was evaluated by conducting a subgroup analysis. The correlation between all the obtained parameters and cycle results (whether gestation occurred, biochemical pregnancy, clinical pregnancy, miscarriage, or live birth) was analysed. Parameters that may effectively predict successful cycle outcome of day 3 and day 5 poor-quality embryo transfers were also evaluated.

### 2.4. Pregnancy Outcomes

Plasma BhCG value above ≥10 IU/l measured on day 14 of embryo transfer was defined as a positive pregnancy result. Pregnancy loss before the gestational week 22 was called a miscarriage, and the birth of a live baby after the gestational week 22 was specified as live birth.

### 2.5. Statistical Method

Data were analysed using IBM SPSS V23 software (IBM, Armonk, NY, USA). Conformity to normal distribution was analysed using the Kolmogorov–Smirnov test. According to groups, Pearson’s Chi-square test, Fisher’s Exact test, and Yates’ Correction were utilised for the categorical data comparison. Mann–Whitney U test was used to compare data that do not distribute normally based on two groups. Binary logistic regression analysis was conducted for the affecting factors, and the Backward: Wald method was used to include the independent variables in the multivariate model. Analysis results were presented as mean ± standard deviation, median (minimum–maximum), and categorical data as frequency (percentage). The significance level was taken as *p* < 0.050.

## 3. Results

**Figure 1:**
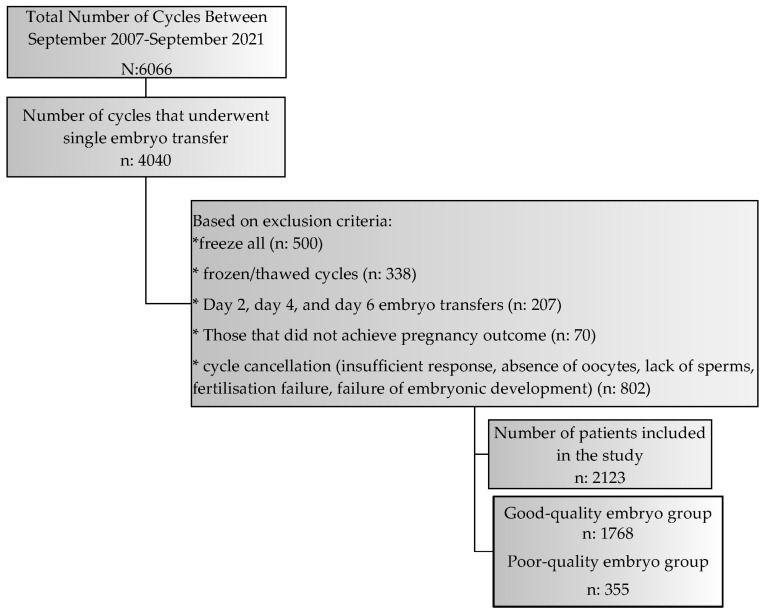
Flow chart of the study group.

In this study, 2123 IVF cycles, divided into two groups with good-quality embryo transfers and poor-quality embryo transfers, were examined. Good-quality embryos were obtained in 1768 of these cycles, and poor-quality embryos were obtained in 355 of these cycles. A flow chart of the study group is shown in Figure 1.

Mean age of the female and male, basal FSH, and E2 levels were higher, and total AFC was lower in the poor-quality embryo group than the good-quality embryo group (*p* < 0.001). When treatment indications were evaluated, a statistically significant difference was found in those with advanced maternal age and unexplained infertility diagnosis. Age-related infertility in 3.2% of the good-quality embryo group and 7.3% of the poor-quality group (*p* < 0.001), and unexplained infertility in 31.2% of the good-quality group and 25.4% of the poor-quality group (*p* = 0.028) was diagnosed.

The total gonadotropin dose used was statistically significantly higher in the cycles of the poor embryo transfer group than the good-quality embryo transfer group (*p* < 0.001). Also, in the group that underwent poor-quality embryo transfer, the E2 level on the trigger day, numbers of oocyte retrieved, numbers of mature oocyte, numbers of oocyte used for ICSI, oocyte quality index, and numbers of 2PN were found to be significantly lower (*p* < 0.001). A significant difference was not observed in other parameters (*p* > 0.050) (Table 1).

Clinical pregnancies rate was 38.3% of the good-quality group and 14.9% of the poor-quality pregnancy group, and the difference was statistically significant (*p* < 0.001). Similarly, the difference regarding the live birth rates between the groups was found to be statistically significant (30.6% in the good-quality embryo group vs. 10.1% in the poor-quality embryo group, *p* < 0.001). Live birth per clinical pregnancy was significantly higher in the good-quality embryo group, and miscarriage per clinical pregnancy was lower (*p* < 0.001). A significant difference was not observed in terms of biochemical pregnancy and miscarriage (*p* > 0.050). Clinical pregnancy was established in 53 patients that underwent transfer in the poor-quality embryo group (14.9%). Of these patients, 36 had live birth (live birth rate per clinical pregnancy 67.9%) (Table 2).

When compared in terms of embryo transfer day, it was found that the clinical pregnancy and live birth rates in the 5th-day transfer were statistically significantly higher compared to the 3rd-day transfer in the good embryo transfer group (46.1% vs. 33% and 37.7% vs. 26.1%, respectively) (Table 3). A statistically significant difference was not observed between day 3 and day 5 embryo transfer in the group that underwent poor-quality embryo transfer regarding clinical pregnancy and live birth rates (*p* > 0.05). However, live birth per clinical pregnancy was higher in embryos transferred on day 3 compared to embryos transferred on day 5 (75% vs. 57.1%, respectively) and miscarriage per clinical pregnancy was lower (25% vs. 42.8%, respectively) (*p* < 0.001) (Table 3).

Factors affecting the hCG positivity in poor-quality embryo groups were examined using binary logistic regression analysis. When analysing the univariate model results, the chance of conception was lower parallel to the increase in total gonadotropin dosage adopted in controlled ovarian hyperstimulation (COH) (OR = 0.997; *p* = 0.049). Whereas, in the multivariate model result, the chance of conception increases by 1416 as the 2PN number rises (*p* = 0.045). Other factors did not have a statistically significant impact on conception (*p* > 0.050). When analysing the factors affecting live births in the poor-quality embryo group, as the total gonadotropin dose used in COH increases, the probability of live birth decreases, as in the result of pregnancy (OR = 0.995; *p* = 0.024). Other factors, however, were not established to have any statistically significant effect on live birth (*p* > 0.050) (Table 4).

## 4. Discussion

This study showed that the clinical pregnancy rate and live birth rate were lower and miscarriage rate was higher in the poor-quality embryo transfer group than in the good-quality embryo transfer group. However, once clinical pregnancy was achieved, 67.9% of patients in the poor-quality embryo transfer group had a live birth. Although clinical pregnancy and live birth rates were significantly higher following day 5 transfers in good-quality embryo transfers, we established that clinical pregnancy rates were similar in day 3 and day 5 transfers of poor-quality embryo transfers but that the chance of live birth was higher in cleavage period embryos compared with blastocyst transfer if clinical pregnancy was accomplished.

Numerous studies in the literature have shown a strong correlation between low clinical pregnancy rates and live birth rates per transfer in morphologically poor-quality embryos [4,5,6,7]. In their study that compared the transfer cycles of 488 patients, Kirilova et al. reported that implantation, clinical pregnancy, and live birth rates of poor-quality embryo transfer cycles were lower compared with cycles with good-quality embryo transfer and that the results did not demonstrate any difference based on whether they were day 3 or day 5 transfer [4]. Although Oron G et al. [5] reported that miscarriage per clinical pregnancy and live birth rate per clinical pregnancy were equivalent between poor- and high-quality embryo transfer groups, a study by Zhu J et al. [6] demonstrated that live birth rate per clinical pregnancy was considerably lower in the poor-quality embryo transfer group compared with a good-quality embryo transfer group and that miscarriage rate per clinical pregnancy was higher. While the previous study [5] combined both cleavage and blastocyst embryos, the latter study [6] evaluated only cleavage embryos and defended the idea that the quality of embryos may affect pregnancy results following the clinical pregnancy during the cleavage stage. Similarly, Akamine et al. [7] combined both groups and found that, in the poor-quality embryo transfer group, the live birth rate per clinical pregnancy was significantly lower, and the miscarriage rate per clinical pregnancy was higher in the good-quality embryo transfer group (69.1% vs. 49.1% and 26% vs. 40.4%). Li M et al., on the other hand, found that approximately 70% (17/22) of infertile patients resulted in live birth following achieving clinical pregnancy (21.6%, 22/102) after low-grade blastocyst transfer, which is generally not preferred for embryo transfer [8].

This study also established a significant difference in good-quality and poor-quality embryo transfer groups in terms of clinical pregnancy and live birth (38.3% vs. 14.9% and 30.6% vs. 10.1%). However, of the poor-quality embryo transfer pregnancies reaching the clinical pregnancy stage, 67.9% resulting in live birth stands out as remarkable. When a comparison is conducted based on transfer day as well, it was observed that the clinical pregnancy and live birth rates via blastocyst transfer in good-quality embryo transfers were significantly higher and that a difference was not found based on transfer day between clinical pregnancy and live birth rates following poor-quality embryo transfers. However, in cleavage-stage embryos, live birth rates per clinical pregnancy were higher in poor-quality blastocyst transfer in the poor-quality embryo group.

Numerous studies have shown that blastocyst transfer theoretically achieved a better synchronisation between the embryonic stage and endometrial receptivity, was more physiological, and accomplished undeniable success rates, specifically in the population with good prognoses [10,11,12,13]. It was explicitly proven in the cleavage stage that the embryo culture strategy was distinctively effective when numerous good-quality embryos were present [12,13]. However, although sufficient data are non-existent, embryo quality during the cleavage stage may not seem to affect clinical results following good-quality blastocyst transfer [10,12]. We established in the literature that the value of foresight was limited on blastocyst formation following day 3 embryo morphology and that only 51% of embryos pre-selected for transfer on day 3 and only 21% of poor-quality embryos achieved blastocysts on day 5 [14]. Another study claimed that grade 3 and 4 embryos had similar rates for progressing to blast but could not foresee blastocyst quality forming [15]. A recent review suggested the view that poor-quality blastocyst transfer should be focused on rather than a poor-quality day 3 embryo and that transfer can be carried out during the cleavage stage only when a single poor-quality embryo is present [16]. Delaying transfer until the blastocyst stage allows selection of higher-quality embryos and enables transfer at the time a naturally fertilized in vivo embryo would have reached the uterine cavity. However, the live birth rate per oocyte retrieval may not improve because fewer embryos are available for transfer due to loss of embryos that did not survive in vitro to day 5. In our study, we did not establish a difference between clinical pregnancy and live birth rates based on transfer day following the poor-quality embryo transfer either. However, in cleavage-stage embryos, live birth rates per clinical pregnancy were higher in poor-quality blastocyst transfer (75% vs, 57.1%). These results give rise to the thought that the success rate would be higher if the patient were to have cleavage-stage transfer instead of waiting until day 5 for patients with poor-quality embryos on day 3 and that this would be a more appropriate approach.

In ART, obtaining as many transferable embryos as possible is critical to having an increased clinical pregnancy rate. And the grounds for attaining transferable embryos is to obtain many normal fertilized oocytes. In this study, when analysing the demographic and COH parameters of the group that received poor-quality embryos, what stands out is that they were of more advanced age compared with the other group, their basal FSH values were higher, and their AFC levels were lower, and also during COH, these patients had fewer oocytes while receiving more gonadotropin. All these factors demonstrate that the total gonadotropin dose used during COH significantly affected live birth as much as pregnancy outcome.

Numerous publications demonstrated that increased numbers of oocytes retrieved improves the live birth rate [17,18,19], and when the effect of obtained numbers of oocytes is analysed, COH may be accepted as a key success factor. A previous study in the literature showed that the higher the Ovarian Sensitivity Index (OSI; the ratio between gonadotropin dosage and numbers of oocyte retrieved), the higher the pregnancy rates. It also suggested that healthy ovaries that respond well to low-dose gonadotropins provide better oocyte quality and fertilization [20].

However, it was suggested that excessive ovarian stimulation has a detrimental effect on oocyte and embryo quality and that this causes embryos with higher aneuploidy rates. Its effects on oocyte and embryo quality were specifically well-characterised in animal models, and it was shown that aggressive stimulation caused a weaker embryo development potential and that it may increase chromosomal anomaly rates and embryo development is negatively impacted in a dose-dependent manner [21,22,23,24,25,26,27,28]. Results of a dose-response study conducted on cattle showed that FSH doses exceeding maximum response caused a reduction in ovarian follicle count per OPU, E2 production, number of collected oocytes, and number of fertilised oocytes [22,23,24,25], resulting in the rise of degenerated embryo count [22], and that it increased aneuploidy compared with mild protocols in rats as well [26]. In addition, it was demonstrated that high dosage caused luteinisation in FSH granulosa cells and that it deteriorated blastocyst development independently of luteinisation [27,28]. As for humans, it was suggested that the euploid oocyte rate was directly proportional to the number of mature oocytes and inversely proportional to the number of gonadotropin units used per mature oocyte obtained [29,30]. Studies conducted with preimplantation genetic screening (PGS) showed that high ovarian response to conventional ovarian stimulation did not raise embryo aneuploidy rates [31,32].

The majority of studies conducted on humans designed to evaluate the effect of gonadotropins on oocyte quality compared distinct gonadotropin doses. When mild and conventional stimulation were compared, a higher rate of good morphological quality embryos was observed in mild protocols [33,34]. Baker et al. analysed the correlation between total gonadotropin dosage and live birth in quite a vast patient series (more than 650,000) that received fresh cycles and observed that gonadotropin dosage as a factor independent of the number of collected oocytes demonstrated a negative correlation with the chance of live birth. They established a 20% decrease in live birth between cycles that received higher doses and those with lower doses [35]. Additionally, the effects of supraphysiological E2 levels or P secretion during high-dose gonadotropin stimulation on endometrium should not be overlooked [36,37]. In this study, too, which supports the results noted above, the rise in total gonadotropin dosage was demonstrated to be a parameter negatively impacting pregnancy and live birth rates.

## 5. Conclusions

Consequently, considering that the obstetric and neonatal outcomes of pregnancies occurring after poor-quality embryo transfers are like good-quality embryos, this could be regarded as an acceptable option in cycles when only poor-quality embryos are available. Patients should be clearly informed that, although the probability of pregnancy is low, when clinical pregnancy is established, there is a high chance of reaching term and having a live birth.

## Figures and Tables

**Table 1:** Patient characteristics and clinical parameters of cycles according to the quality.

		Good Quality n: 1768	Poor Quality n: 355	*p* Value
Female age (years)		29.4 ± 4.5	31.0 ± 5.3	<0.001 *
Body mass index (kg/m^2^)		26.2 ± 5.0	26.1 ± 5.1	0.703 *
Number of cycles		1.5 ± 0.8	1.7 ± 1.0	<0.001 *
Basal D3 FSH (IU/L)		8.2 ± 21.2	8.8 ± 6.1	<0.001 *
Basal D3 E2 (pg/mL)		48.1 ± 32.4	50.6 ± 34.6	0.021 *
AMH (ng/mL)		3.0 ± 3.5	2.7 ± 3.0	0.376 *
Duration of infertility (months)		62.1 ± 43.4	63.5 ± 46.9	0.906 *
Numbers of total antral follicle		14.0 ± 8.4	12.4 ± 8.2	<0.001 *
Male age (years)		32.7 ± 4.9	34.5 ± 7.3	<0.001 *
Total progressive motile sperm count (n)		22,133.4 ± 41,165.3	24,006.2 ± 48,095.5	0.336 *
Sperm morphology: normal rate (%)		8.2 ± 4.3	6.7 ± 7.5	0.806 *
Indication of treatment (%)				
Male factor		733 (41.5)	140 (39.4)	0.480 **
Tubal factor		127 (7.2)	19 (5.4)	0.259 ***
Diminished ovarian reserve		243 (29.3)	71 (35.9)	0.072 **
Advanced female age		56 (3.2)	26 (7.3)	<0.001 ***
Hormonoovulatory dysfunction		313 (17.7)	57 (16.1)	0.455 **
Unexplained infertility		552 (31.2)	90 (25.4)	0.028 **
Severe pelvic adhesion		11 (0.6)	2 (0.6)	1000 ^†^
Endometriosis		102 (5.8)	23 (6.5)	0.604 **
Gnrh protocol (%)				
Microdose flare-up		99 (5.6)	16 (4.5)	0.462 **
Antagonist		984 (55.7)	209 (58.9)	
Long luteal		685 (38.7)	130 (36.6)	
Duration of ovarian stimulation (n)		9.9 ± 1.7	10.0 ± 1.8	0.441 *
Total gonadotropin dose (IU)		2187.1 ± 897.0	2414.8 ± 1038.2	<0.001 *
Trigger (%)				
Hcg		1585 (89.6)	322 (90.7)	0.549 **
Agonist		183 (10.4)	33 (9.3)	
Trigger Day	E2 level (pg/mL)	2433.7 ± 1629.4	2197.3 ± 1470.2	0.027 *
	≥17 mm follicle (n)	3.3 ± 2.6	3.3 ± 2.6	0.671 *
	15–17 mm follicle (n)	3.8 ± 3.1	3.3 ± 2.7	0.001 *
	10–14 mm follicle (n)	6.0 ± 4.8	4.9 ± 4.2	<0.001 *
	Endometrial thickness (mm)	10.1 ± 2.0	9.8 ± 2.1	0.072 *
Number of oocytes retrieved		11.9 ± 7.3	10.3 ± 6.7	<0.001 *
Number of mature oocytes		8.9 ± 5.6	7.6 ± 5.3	<0.001 *
Number of oocytes used for ICSI		9.4 ± 5.7	8.2 ± 5.5	<0.001 *
Oocyte quality index		5.1 ± 0.8	5.0 ± 0.8	0.001 *
Numbers of 2PN		4.8 ± 3.6	3.6 ± 2.7	<0.001 *
Blastocyst transfer (n)		0.33 ± 0.48	0.33 ± 0.49	0.980 *
Embryo transfer Day (%)				
Day 3 embryo transfers		1055 (59.7)	221 (62.3)	0.365 **
Day 5 embryo transfers		713 (40.3)	134 (37.7)	

* Mann–Whitney U test ** Chi-square test, *** Yates’ Correction, ^†^ Fisher’s Exact test.

**Table 2:** Clinical outcomes of poor- and good/fair-quality embryos.

	Good Quality n: 1768	Poor Quality n: 355	*p* Value
No pregnancy	992 (56.1)	283 (79.7)	<0.001
Biochemical pregnancy	99 (5.6)	19 (5.4)	NS
Clinical pregnancy	677 (38.3)	53 (14.9)	<0.001
Miscarriage	136 (7.7)	17 (4.8)	NS
Live birth	541 (30.6)	36 (10.1)	<0.001
Live Birth per Clinical Pregnancy	541/677 (79.9)	36/53 (67.9)	<0.001
Miscarriage per Clinical Pregnancy	136/677 (20)	17/53 (32)	<0.001

Chi-square test, NS: not statistically significant.

**Table 3:** Clinical outcomes of the poor- and good/fair-quality embryo transfers in patients on day 3 or day 5 embryo transfers.

		Day 3 Transfers (n: 1376)	Day 5 Transfers (n: 847)	*p* Value
Good quality	No pregnancy	655 (62.1)	337 (47.3)	<0.001 *
	Biochemical pregnancy	52 (4.9)	47 (6.6)	NS *
	Clinical pregnancy	348 (33)	329 (46.1)	<0.001 *
	Miscarriage	73 (6.9)	63 (8.8)	NS *
	Live birth	275 (26.1)	266 (37.3)	<0.001 *
	Live Birth per Clinical Pregnancy	275/348 (79)	266/329 (80.8)	NS *
	Miscarriage per Clinical Pregnancy	73/348 (20.9)	63/329 (19.1)	NS *
Poor quality	No pregnancy	180 (81.4)	103 (76.9)	NS **
	Biochemical pregnancy	9 (4.1)	10 (7.5)	NS **
	Clinical pregnancy	32 (14.5)	21 (15.7)	NS **
	Miscarriage	8 (3.6)	9 (6.7)	NS **
	Live birth	24 (10.9)	12 (9)	NS **
	Live Birth per Clinical Pregnancy	24/32 (75)	12/21 (57.1)	<0.001 **
	Miscarriage per Clinical Pregnancy	8/32 (25)	9/21 (42.8)	<0.001 **

* Chi-square test, ** Yates’ Correction, NS: not statistically significant.

**Table 4:** Analysis of factors affecting hCG positivity and live birth in poor-quality embryos.

		hCG Positivity		Live Birth	
		OR (95% CI)	*p* Value	OR (95% CI)	*p* Value
Indication of treatment (%)					
Male factor		0.972	0.915	1.109	0.773
Tubal factor		0.447	0.289	0.478	0.479
Diminished ovarian reserve		0.871	0.690	0.96	0.920
Age-related infertility		0.308	0.116	3.243	0.116
Hormonoovulatory dysfunction		0.945	0.874	0.951	0.916
Unexplained infertility		0.977	0.939	0.826	0.649
Severe pelvic adhesion		3.972	0.332	0.252	0.332
Endometriosis		1.099	0.857	0.835	0.813
Female age (years)		0.976	0.339	0.945	0.111
Body Mass Index (kg/m^2^)		1.024	0.363	1.048	0.158
Numbers of cycles		0.906	0.484	0.932	0.704
Basal D3 FSH (IU/L)		0.963	0.274	0.934	0.211
Basal D3 E2 (pg/mL)		0.995	0.368	0.998	0.715
AMH (ng/mL)		1.001	0.988	1.073	0.321
Duration of infertility (months)		1	0.904	0.997	0.497
Numbers of total antral follicle		1.015	0.352	1.022	0.280
Male age (years)		0.986	0.486	0.97	0.279
Total progressive motile sperm count		1	0.928	1	0.663
Sperm morphology: normal rate		1.01	0.655	1.039	0.133
Protocol (Reference: long luteal)					
Microdose flare-up		1.112	0.655	1.835	0.440
GnRH antagonist		0.955	0.646	1.012	0.966
Duration of ovarian stimulation		0.936	0.383	0.903	0.330
Total gonadotropin dose (IU)		0.997	0.049	0.995	0.024
Trigger		1.064	0.889	1.676	0.321
Trigger day	E2 level (pg/mL)	1	0.376	1	0.423
	≥17 mm follicle	1.062	0.223	0.987	0.853
	15–17 mm follicle	1.012	0.809	0.936	0.351
	10–14 mm follicle	1.043	0.150	1.043	0.269
	Endometrial thickness (mm)	1.103	0.155	1.072	0.453
Numbers of oocyte retrieved		1.032	0.090	1.035	0.152
Numbers of mature oocyte		1.037	0.120	1.035	0.256
Number of oocytes used for ICSI		1.036	0.122	1.032	0.295
Oocyte quality index		0.905	0.537	0.995	0.980
Numbers of 2PN		1.416	0.045	1.097	0.111
Blastocyst transfer		1.006	0.983	0.628	0.268
Transfer Day		1.149	0.299	0.899	0.565

Backward: Wald method was used to include the independent variables in the multivariate model.

## Data Availability

Data can be made accessible upon demand.

## Abbreviations

IVF: In Vitro Fertilization; ART: Assisted Reproductive Techniques; SET: Single Embryo Transfer; FSH: Follicle Stimulating Hormone; E2: Estradiol; AFC: Antral Follicle Count; ICSI: Intracytoplasmic Sperm Injection; 2PN: 2Pronuclear; hCG: Human Chorionic Gonadotropin; COH: Controlled Ovarian Hyperstimulation; OSI: Ovarian Sensitivity Index; OPU: Oocyte pick-up; PGS: Preimplantation Genetic Screening; BMI: Body Mass Index; AMH: Anti-Mullerian Hormone; TPMSC: Total Progressive Motile Sperm Count; GnRH: Gonadotropin-Releasing Hormone.
